# Factors Associated with the Occurrence of Cardiac Arrest after Emergency Tracheal Intubation in the Emergency Department

**DOI:** 10.1371/journal.pone.0112779

**Published:** 2014-11-17

**Authors:** Won Young Kim, Myoung Kwan Kwak, Byuk Sung Ko, Jae Chol Yoon, Chang Hwan Sohn, Kyoung Soo Lim, Lars W. Andersen, Michael W. Donnino

**Affiliations:** 1 Department of Emergency Medicine, Ulsan University College of Medicine, Asan Medical Center, Seoul, Korea; 2 Department of Emergency Medicine, Seoul Medical Center, Seoul, Korea; 3 Department of Emergency Medicine, Chonbuk National University Hospital, Jeonju, Korea; 4 Department of Emergency Medicine, Beth Israel Deaconess Medical Center, Boston, Massachusetts, United States of America; 5 Department of Anesthesiology, Aarhus University Hospital, Aarhus, Denmark; Temple University, United States of America

## Abstract

**Objectives:**

Emergency tracheal intubation has achieved high success and low complication rates in the emergency department (ED). The objective of this study was to evaluate the incidence of post-intubation CA and determine the clinical factors associated with this complication.

**Methods:**

A matched case-control study with a case to control ratio of 1∶3 was conducted at an urban tertiary care center between January 2007 and December 2011. Critically ill adult patients requiring emergency airway management in the ED were included. The primary endpoint was post-intubation CA, defined as CA within 10 minutes after tracheal intubation. Clinical variables were compared between patients with post-intubation CA and patients without CA who were individually matched based on age, sex, and pre-existing comorbidities.

**Results:**

Of 2,403 patients who underwent emergency tracheal intubation, 41 patients (1.7%) had a post-intubation CA within 10 minutes of the procedure. The most common initial rhythm was pulseless electrical activity (78.1%). Patients experiencing CA had higher in-hospital mortality than patients without CA (61.0% vs. 30.1%; p<0.001). Systolic hypotension prior to intubation, defined as a systolic blood pressure ≤90 mmHg, was independently associated with post-intubation CA (OR, 3.67 [95% CI, 1.58–8.55], p = 0.01).

**Conclusion:**

Early post-intubation CA occurred with an approximate 2% frequency in the ED. Systolic hypotension before intubation is associated with this complication, which has potentially significant implications for clinicians at the time of intubation.

## Introduction

Successful emergency airway management is an essential component of the modern practice of emergency medicine [1]. Rapid sequence intubation, a technique for airway management in the emergency department (ED), is has become an important procedure [2]–[5]. Previous reports have shown that emergency airway management in the ED has a high success rate with few complications [2], [4]. Nonetheless, a number of immediate complications after emergency airway management have been reported including failed intubation, hypoxemia, hypotension, arrhythmia, and even cardiac arrest (CA) [6]–[13]. Emergency airway management in the ED can be more fraught with complications related to hemodynamic alterations and other airway-related complications more than in the operating room where most intubations are performed under controlled conditions [5], [14], [15]. Furthermore, the subject of CA as a complication of emergency tracheal intubation outside the operation room has not been well-reported in the literature [15]–[17].

The objective of this study was to evaluate the incidence of post-intubation CA and to determine the clinical factors associated with this complication.

## Methods

### Study design and population

This was a retrospective matched case-control study with a case to control ratio of 1∶3 that was conducted at a 2,800-bed, university-affiliated, tertiary referral center in Seoul, Korea. This study was approved by the Institutional Review Board of the Ethics Committee of the Asan Medical Center, which waived the requirement of informed consent for this retrospective review of anonymized medical records. Between January 2007 and December 2011, 2,403 adult (18 years or older) non-traumatic, critically ill patients had emergency airway management in our ED. A total of 41 patients who developed post-intubation CA, defined as CA within 10 minutes after successful intubation, were identified. Each case was matched to three adult controls, who did not develop CA after intubation in the ED, according to the following criteria: (1) age within 2 years; (2) same gender; and (3) same pre-existing comorbidities such as hypertension or chronic obstructive lung disease. In this manner, 123 controls were matched to 41 patients who developed CA after emergency tracheal intubation, for a total study population of 164.

### Data collection and clinical setting

The clinical and demographic characteristics of all study patients, including age, gender, past medical history, and pre-intubation vital signs were retrospectively retrieved from electronic hospital records. During the study period, a board-certified emergency medicine physician covered the 24-hour airway management in the ED. Emergency physicians used their clinical judgment to determine the approach to airway management, including the intubation route, pre-medications, and sedatives. Ventilation with 100% oxygen for 2 to 4 minutes before and between intubation attempts of greater than 30 seconds was the standard practice. Following intubation, the airway manager completed a questionnaire that outlined the procedural details, hemodynamic alterations, and any complications. Intubation procedural data included the number of intubation attempts, routes of intubation, level of consciousness, medications administered for patient preparation, drug dosages, pre-intubation hemodynamic data, and complications.

## Statistical Analysis

The data in this study are presented as the mean ± standard deviation or median with the interquartile range (IQR) for continuous variables and as absolute or relative frequencies for categorical variables. Patients who developed CA within 10 minutes of intubation were compared with those who did not. Unpaired Student’s *t* tests or Mann-Whitney *U* tests were used to compare continuous variables, and chi-square tests were used for categorical variables. Associated factors for the development of post-intubation CA were assessed by univariate analysis, and variables that were statistically significant (p<0.20) in the univariate analysis were included in the multivariate analysis. Stepwise modeling was used to screen potential variables for inclusion in the final model. The results of the multivariate logistic regression analysis were reported as odds ratios (OR) and 95% confidence intervals (CI). A p value≤0.05 was considered to be statistically significant. All statistical analyses were performed using SPSS for Windows version 18.0 (SPSS Inc., Chicago, IL).

## Results

### Incidence and demographics

**Figure 1:**
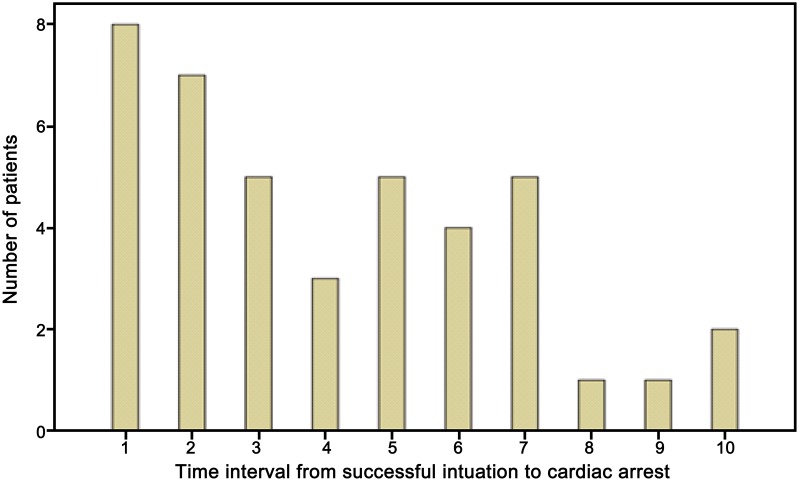
Time interval from successful intubation to cardiac arrest for study subjects.

Of the 2,403 patients who underwent an emergency tracheal intubation at our institution during the study period, a total of 41 post-intubation CA occurred in the ED, for an overall incidence of 1.7%. The affected patients ranged in age from 36 to 85 years (mean, 64.5 years) and 108 (65.9%) were male. The median time from a successful intubation to CA in these cases was 6.0 minutes (IQR, 2.5–8.5). A total of 68.3% post-intubation CA occurred within 5 minutes after successful intubation (see Figure 1). Additional demographic characteristics and the clinical features of the patients in each group are summarized in Table 1.

**Table 1:** Demographic and clinical characteristics of the study subjects.

	Cardiac arrest group(*n* = 41)	Control group(*n* = 123)	p-value
Demographics			
Age, years	64.0±12.1	64.7±11.1	0.72
Male	27 (65.9)	81 (65.9)	1.00
Body weight, kg	60.7±14.5	60.3±10.7	0.92
Initial vital signs†			
Systolic blood pressure, mmHg	98.3±38.2	125.6±35.9	<0.01
Systolic blood pressure ≤90	17 (41.5)	18 (14.6)	<0.01
Diastolic blood pressure, mmHg	59.0±24.2	71.5±21.9	0.01
Pulse rate, per minute	101.6±33.3	102.6±33.1	0.87
Respiratory rate, per minute	26.0±11.0	26.7±9.6	0.71
Body temperature, °C	36.6±1.2	36.9±0.92	0.14
Oxygen saturation, %	86.9±11.0	87.7±15.1	0.77
Mean arterial pressure, mmHg	72.1±27.7	89.5±24.5	<0.01
Mean arterial pressure ≤65	16 (39.0)	19 (15.4)	<0.01
Shock index	1.1±0.4	0.9±0.4	0.03
Mental state			0.69
Alert	17 (41.5)	63 (51.2)	
Verbal response	5 (12.2)	12 (9.8)	
Pain response	9 (22.0)	26 (21.1)	
No response	10 (24.4)	22 (17.9)	
Vasopressor use	9 (22.0)	12 (9.8)	0.06
Reason for Emergency Room			
Medical	31 (75.6)	83 (72.4)	0.55
Reason for intubation			0.72
Acute respiratory failure	23 (56.1)	71 (57.7)	
Shock	7 (17.1)	13 (10.6)	
Altered mental status	5 (12.2)	17 (13.8)	
Others	6 (14.6)	22 (17.9)	
Number of intubation attempts			0.63
1	36 (87.8)	105 (85.4)	
2	3 (7.3)	15 (12.2)	
≥3	2 (4.9)	3 (2.4)	
Survival discharge	15 (36.6)	86 (69.9)	<0.01

Values are expressed as the mean ± SD or n (%).

† Vital signs are the measured values directly before tracheal intubation.

### Method of emergency airway management

The medical profile varied widely but acute respiratory failure was the most common clinical indication for emergency airway management (56.1%). More people in the CA arrest group underwent intubation due to shock but this did not reach statistical significance (Table 1). The types and dosages of sedative, opioid, and neuromuscular drugs used are shown in Table 2. Only nine patients (5.5%) did not receive parenteral medication. Pretreatment drugs were used in 129 patients (78.7%): fentanyl in 121 patients and lidocaine in 26 patients. Nearly all of the patients (92.1%) received sedative medications, including etomidate in 131 patients, midazolam in 15 patients, ketamine in 5 patients, propofol in 3 patients, and other types in 9 patients.

**Table 2:** Incidence of Use of Each Drug for Endotracheal Intubation.

	Cardiac arrest group (*n* = 41)	Control group (*n* = 123)	p-value
Pretreatment drugs			
Fentanyl	28 (68.3)	93 (75.6)	0.41
Dose, ng/kg	1.5 (1.3–2.0)	1.7 (1.5–2.0)	0.12
Lidocaine	6 (14.6)	20 (16.3)	1.00
Dose, mg/kg	1.4 (0.7–2.0)	1.5 (1.0–1.9)	0.83
Neuromuscular blocking drugs			
Succinylcholine	4 (9.8)	17 (13.8)	0.60
Dose, mg/kg	1.1 (0.6–1.2)	1.0 (0.7–1.0)	0.88
Hypnotics			
Etomidate	30 (73.2)	101 (82.1)	0.26
Dose, mg/kg	0.2 (0.2–0.4)	0.3 (0.2–0.4)	0.20
Midazolam	5 (12.2)	10 (8.1)	0.53
Ketamine	0 (0.0)	5 (4.1)	0.70
Propofol	0 (0.0)	3 (2.4)	0.83
Others	2 (4.9)	7 (5.7)	0.90

Values are expressed as median with interquartile range or as n (%).

### Characteristics of post-intubation CA

Of the 41 CA cases associated with intubation, 32 were characterized by a progressive decline in heart rate, leading to pulseless electrical activity, which was the most common initial arrest rhythm (78.1%). Thirty-six patients were successfully intubated on the first attempt. However, 5 of the 41 CA patients required more than two intubation attempts. The main outcomes of the 41 post-intubation CA patients were that 31 patients (75.6%) had a sustained return of spontaneous circulation, 16 patients (39.0%) survived to discharge, and 6 patients (14.6%) had a good neurologic outcome, defined as a cerebral performance category score of 1 or 2. Patients experiencing CA had higher in-hospital mortality (61.0% vs. 30.1%; p<0.001) and post-intubation CA was associated with in-hospital mortality (OR, 3.71 [95% CI, 1.58–8.70]; p = 0.01).

### Factors associated with post-intubation CA

Mental status, reason for intubation, and number of intubation attempts were not significantly different between the groups. However, systolic/diastolic blood pressure (BP) and mean arterial pressure (MAP) were significantly lower in the CA group than the control group. Furthermore, our patients with post-intubation CA had a significantly higher incidence of systolic hypotension, defined as a systolic BP ≤90 mmHg (41.5% vs. 14.6%; p<0.01) and hypotension, defined as a MAP≤ 65 mmHg (39.0% vs. 15.4%; p<0.01). Multivariate analysis showed that systolic hypotension before intubation was independently associated with post-intubation CA (3.67 [95% CI, 1.58–8.55]; p = 0.01).

## Discussion

This study evaluated the incidence of post-intubation CA and identified factors associated with this complication in the ED. We found that the incidence of early post-intubation CA (within 10 minutes), a major complication of emergency tracheal intubation associated with in-hospital mortality, occurred with an approximate 2% frequency in the ED. This may be higher than commonly appreciated. In addition, we found that pre-intubation BP variables, specifically a systolic BP ≤90 mmHg, were independently associated with post-intubation CA.

A number of previous studies have reported major complication rates associated with endotracheal intubation. While the rate of complications during intubations in the operating room is generally very low (less than 1 per 10,000 cases), much higher rates are reported in the ED, intensive care unit, and on in-patient wards [10], [15], [18]–[22]. In a multicenter study of almost 9,000 intubations in the ED, Walls et al. [18] reported an overall complication rate of about 12%, although the rate of CA after intubation was reported at only 0.6%. In contrast, Heffner et al. [23] found a post-intubation CA rate of 4.1% within 35 minutes and 2.4% within 10 minutes of the intubation, which is similar to our current findings of 1.7% within 10 minutes of the intubation.

However, Heffner et al. [23] recently found in a study from a large American medical center that a higher pre-intubation shock-index (defined as heart rate divided by systolic BP) and a higher patient weight were independently associated with post-intubation CA. The importance of cardiovascular deterioration before intubation is not unexpected. Our present data show that systolic hypotension (BP ≤90 mmHg), which is easy to monitor in real field conditions, is independently associated with post-intubation CA (3.67 [95% CI, 1.58–8.55]; p = 0.01). Although our current study included almost three times as many patients experiencing CA as previous reports, it was difficult to identify the optimal cut-off value that was accurately associated with post-intubation CA. However, our present findings provide some insights into this critical complication at the time of intubation.

While the causal pathway for early post-intubation CA cannot be determined from the current or previous studies, a number of potential mechanisms could account for the association between pre-intubation hypotension and post-intubation CA. Emergency intubation can cause hypotension, potentially as a consequence of the actual intubation (sympatholytic stimulus), mechanical ventilation (positive pressure ventilation with subsequent decrease in venous return), and/or the induction agents used [24]–[26]. The intubation may have exacerbated pre-existing hypotension leading to a progressive decline in BP and subsequently pulseless electrical activity arrest–the most common initial rhythm found in our current study. Alternately, the association between pre-intubation hypotension and post-intubation CA may be an indicator of a sicker patient population who would have deteriorated to CA irrespective of the intubation and other interventions. Further research is needed to clarify this point.

The association between pre-intubation hypotension and post-intubation CA raises the question of whether treatment of pre-intubation hypotension by fluid resuscitation and/or vasopressors can decrease the rate of post-intubation CA. To our knowledge, no such studies have been carried out in an ED setting. However, Jaber et al. [6] have previously conducted a two-phase, multicenter trial assessing the effect of an intubation bundle checklist on intubation complications in the intensive care unit. This intervention consisted of pre-intubation checks (including provision of a fluid bolus if appropriate), intubation best practices, and post-intubation checks. This intervention was associated with a significantly lower rate of life-threatening complications and a significantly lower rate of mild-to-moderate complications. They also found a lower rate of post-intubation CA; however, this finding did not reach statistical significance. Whether these findings are translatable to the ED setting remains to be determined. Our current study, along with other previous reports [23], [24], [27], provides the incentive for this to be investigated in the future.

## Limitations

The main limitations of the present study are its retrospective nature and the individual-matched case-control design. The low number of post-intubation CA patients (*n* = 41) might have contributed to the reduced statistical power of some results, although this is the largest sample size reported to date. Another potential concern is the use of matched controls. Although we matched patients with control subjects of similar age, gender, and comorbidities to create a more homogenous population, a potential selection bias might have been introduced. Furthermore, we cannot assess the association between these variables and the occurrence of post-intubation CA as they were used for matching. More than 70% of the patients in out cohort received etomidate as sedation which limits our ability to assess the relationship between drug choice and progression to cardiac arrest. We evaluated CA within 10 minutes of intubation and the incidence of this complication may be further increased when considering a longer time interval. Finally, this was also a single-center study, and our results may not be easily applicable to other settings.

## Conclusions

Early, post-intubation CA (within 10 minutes of intubation) occurred with an approximate 2% frequency in the ED. Pre-intubation hypotension is associated with the occurrence of CA. Further research is needed to clarify whether optimization of hypotension before intubation could lower the rate of post-intubation CA.
